# Interventions to Improve Adherence in Patients with Immune-Mediated Inflammatory Disorders: A Systematic Review

**DOI:** 10.1371/journal.pone.0145076

**Published:** 2015-12-16

**Authors:** Fanny Depont, Francis Berenbaum, Jérome Filippi, Michel Le Maitre, Henri Nataf, Carle Paul, Laurent Peyrin-Biroulet, Emmanuel Thibout

**Affiliations:** 1 SC Partners, Paris, France; 2 Sorbonne University, UPMC Université Paris 06, UMRS 938, DHU i2B, Department of Rheumatology, AP–HP, Saint-Antoine Hospital, Paris, France; 3 Department of Gastroenterology, Archet 2 Hospital, Nice, France; 4 60 rue du Tour de Ville, 14880 Colleville-Montgomery, France; 5 François Quesnay Hospital, Mantes la Jolie, France; 6 Department of Dermatology, Paul Sabatier University, Toulouse, France; 7 Inserm U954 and Department of Gastroenterology, Université de Lorraine, Vandoeuvre-les-Nancy, France; 8 AbbVie, Rungis, France; Renal Division, Peking University First Hospital, CHINA

## Abstract

**Background:**

In patients with immune-mediated inflammatory disorders, poor adherence to medication is associated with increased healthcare costs, decreased patient satisfaction, reduced quality of life and unfavorable treatment outcomes.

**Objective:**

To determine the impact of different interventions on medication adherence in patients with immune-mediated inflammatory disorders.

**Design:**

Systematic review.

**Data sources:**

MEDLINE, EMBASE and Cochrane Library.

**Study eligibility criteria for selecting studies:**

Included studies were clinical trials and observational studies in adult outpatients treated for psoriasis, Crohn’s disease, ulcerative colitis, rheumatoid arthritis, spondyloarthritis, psoriatic arthritis or multiple sclerosis.

**Study appraisal and synthesis methods:**

Intervention approaches were classified into four categories: educational, behavioral, cognitive behavioral, and multicomponent interventions. The risk of bias/study limitations of each study was assessed using the GRADE system.

**Results:**

Fifteen studies (14 clinical trials and one observational study) met eligibility criteria and enrolled a total of 1958 patients. Forty percent of the studies (6/15) was conducted in patients with inflammatory bowel disease, half (7/15) in rheumatoid arthritis patients, one in psoriasis patients and one in multiple sclerosis patients. Seven out of 15 interventions were classified as multicomponent, four as educational, two as behavioral and two as cognitive behavioral. Nine studies, of which five were multicomponent interventions, had no serious limitations according to GRADE criteria. Nine out of 15 interventions showed an improvement of adherence: three multicomponent interventions in inflammatory bowel disease; one intervention of each category in rheumatoid arthritis; one multicomponent in psoriasis and one multicomponent in multiple sclerosis.

**Conclusion:**

The assessment of interventions designed for increasing medication adherence in IMID is rare in the literature and their methodological quality may be improved in upcoming studies. Nonetheless, multicomponent interventions showed the strongest evidence for promoting adherence in patients with IMID.

## Introduction

Immune-mediated inflammatory disorders (IMIDs) refer to a group of chronic diseases involving an immune response that is inappropriate or excessive, and is caused, signified, or accompanied by dysregulation of the body’s normal cytokine milieu [1]. IMIDs bring together conditions such as inflammatory bowel diseases (IBD) including Crohn’s disease (CD) and ulcerative colitis (UC), psoriasis (PS), multiple sclerosis (MS) and rheumatologic conditions (RC) including rheumatoid arthritis (RA), spondyloarthritis (SpA), and psoriatic arthritis (PsA).

In chronic diseases such as asthma, diabetes or hypertension, up to 30% of physician prescriptions are never filled and about 50% of medications are not taken as prescribed in chronic diseases [2–4]. Similarly, poor adherence to medication is a challenge in clinical practice in patients with IMID [5–7]. In such patients, poor adherence was associated with increased healthcare costs [7], decreased patient satisfaction, reduced quality of life and poor treatment outcomes [5, 8, 9]. These data illustrate the need for efficient interventions to improve medication adherence in IMID patients.

Several intervention studies have been conducted to improve adherence to treatment in IMID patients, including information about disease [10], medication reminders using pill-box or mobile phone [11] and motivational interview [8]. However, there was a high level of heterogeneity in study methods as well as little consistency in their conclusions [12–15], which does not allow to draw clear conclusions about the interventions aimed to improve medication adherence in these conditions.

Using a standardized evaluation process, this systematic review aimed to identify the most suitable interventions to improve medication adherence in patients with IMID, according to the four categories proposed by Greenley et al. [16].

## Methods

This systematic review was conducted according to the recommendations presented in the PRISMA Statement [17]. Assistance from clinical experts in dermatology, gastroenterology, and rheumatology was obtained at all stages of protocol generation and implementation. No institutional review board approval was requested because there was no direct involvement of patients. Study protocol is available upon request.

### Study selection, eligibility criteria

Interventions intended to improve adherence with prescribed medications in adult outpatients treated for CD, UC, PS, RA, SpA, PsA, MS were assessed (Table 1).

**Table 1:** Study inclusion and exclusion criteria.

Category	Inclusion criteria	Exclusion criteria
**Population**	Adults of 19 years and over, treated with systemic medications for one of the conditions of interest	- Children younger than 18 years (no adult in the study or outcome of interest not stratified by child/adult)
		- Patients administered medications at hospital
		- Patients taking over-the counter medications not prescribed by a physician
**Conditions of interest**	Psoriasis, Crohn’s disease, ulcerative colitis, rheumatoid arthritis, spondyloarthritis, psoriatic arthritis, multiple sclerosis	- All other conditions
**Geographic area**	Europe and United States	All other countries
**Period**	From January 1990 to December 2013	Before 1990
**Length of follow-up**	No limit	-
**Settings**	Outpatient care setting	Institutional settings (e.g. Inpatient care, nursing home, prisons)
**Interventions**	Any intervention intended to improve adherence with prescribed medications	- Interventions intended to improve primary prevention measures (e.g. diet, physical exercise, lifestyle changes)
		- Intervention assessing change in taking medications (e.g. taken once daily versus twice daily)
		- Policy intervention (e.g. effect of a health policy on adherence)
**Outcomes of interest**	- Primary outcome: adherence to medication	
	- Secondary outcomes: Clinical efficacy criteria (e.g. disease activity), quality of life, costs	
**Publication language**	English	All other language
**Type of study**	- Original research including clinical trials, observational studies with or without statistically significant improvement in medication adherence	- Case series, case reports, non systematic review, editorials, letters to the editor.
	- Additional relevant studies manually identified in systematic review	- Number of included subject < 30
		- Articles rated high risk of bias (very serious limitations)

### Data sources and searches

To identify relevant articles, targeted literature searches were conducted in MEDLINE, EMBASE and Cochrane Library from January 1990 to December 2013. For each condition of interest, Medical Subject Headings (MesH) and text terms related to adherence and interventions were identified. Details on the MEDLINE search strategy are presented in Table 2.

**Table 2:** Medline search strategy.

**MeSH terms**
1. “interventions studies” OR "disease management" OR "self care" OR "physician-patient relations*" OR "text messaging"
2. "patient compliance" OR "medication adherence"
3. "colitis, ulcerative" OR "crohn disease"
4. "arthritis, rheumatoid" OR "spondylitis, ankylosing" OR "arthritis, psoriatic"
5. -
6. "multiple sclerosis"
**Text terms [All Fields]**
1. "intervention(s)” OR "patient support program" OR "internet" OR "cellular phone" OR "mobile phone" OR "behavioral change techniques" OR "motivational interviewing" OR "psychological support" OR "personalized intervention" OR "personalization"
2. "compliance" OR “adherence” OR “persistence” OR “consistency”
3. "crohn" OR "crohn disease" OR "inflammatory bowel disease"
4. "rheumatoid arthritis" OR "ankylosing spondylitis" OR "psoriatic arthritis”
5. "psoriasis"
6. "multiple sclerosis"
**Search strategy**
1. 1 AND 2 AND 3
2. 1 AND 2 AND 4
3. 1 AND 2 AND 5
4. 1 AND 2 AND 6

The grey literature was reviewed post-hoc (opengrey.eu and greylit.org) but no references related to the subject was found. We also searched relevant citations manually in the reference list of pertinent reviews.

Two trained reviewers screened independently each title and abstracts (FD and SR). All titles selected by at least one reviewer went on full-text review. Conflicts were resolved by discussion and consensus. Clinical experts also reviewed the search strategy.

### Data extraction and quality assessment

A trained reviewer extracted data of interest from each study that met the inclusion criteria and summarized them in a structured table. The summarized studies were checked for completeness and accuracy by a second reviewer.

To estimate the magnitude of adherence (size of intervention effect), we calculated the relative risk (RR), which is the ratio of the proportion of patients improving in the intervention group divided by the proportion of patients improving in the control group. RR is easy to interpret and consistent with the way clinicians generally think. [18].

One reviewer (FD) assessed the risk of bias/study limitations of each study using the GRADE system [19]. The five following criteria were examined for clinical trials: 1) Lack of allocation concealment; 2) Lack of blinding; 3) Incomplete accounting of patients and outcome events; 4) Selective outcome reporting bias; 5) Other limitations such as use of non-validated measures of adherence. The risk of bias was quoted: *(i)“no serious limitations”* if there was a low risk of bias for all these key criteria; *(ii) “serious limitations”* if there was crucial limitation for one criterion or some limitations for multiple criteria sufficient to lower the confidence in the estimate of effect; *(iii) “very serious limitations”*. Studies with a very serious limitation quotation were excluded from the review.

Post-hoc study power was calculated from the available data presented in the publication with an online tool [20].

### Data analysis—Classification of interventions

A qualitative analysis of extracted data was performed. Intervention approaches were classified into four categories: educational, behavioral, cognitive behavioral, and multicomponent intervention according to Greenley et *al* [16].

*Educational interventions* aim to enhance patient knowledge of disease and symptoms, the benefits and mechanisms of action of the medication regimen, the consequences of non- adherence and potential side effects of treatment (example: individual or group educational sessions),

*Behavioral interventions* promote the act of medication taking and/or reinforce adherence by providing incentives for medication taking (example: text message sending, motivational interview),

*Cognitive behavioral interventions* enhance adherence by altering thinking patterns that contribute to non-adherence while also establishing behavioral patterns that support adherence using aforementioned behavioral strategies (example: problem-solving skills training),

*Multicomponent interventions* use multiple strategies to enhance adherence including educational, behavioral, cognitive behavioral, motivational and/or support provision strategies.

## Results

### Selected studies

**Fig 1:**
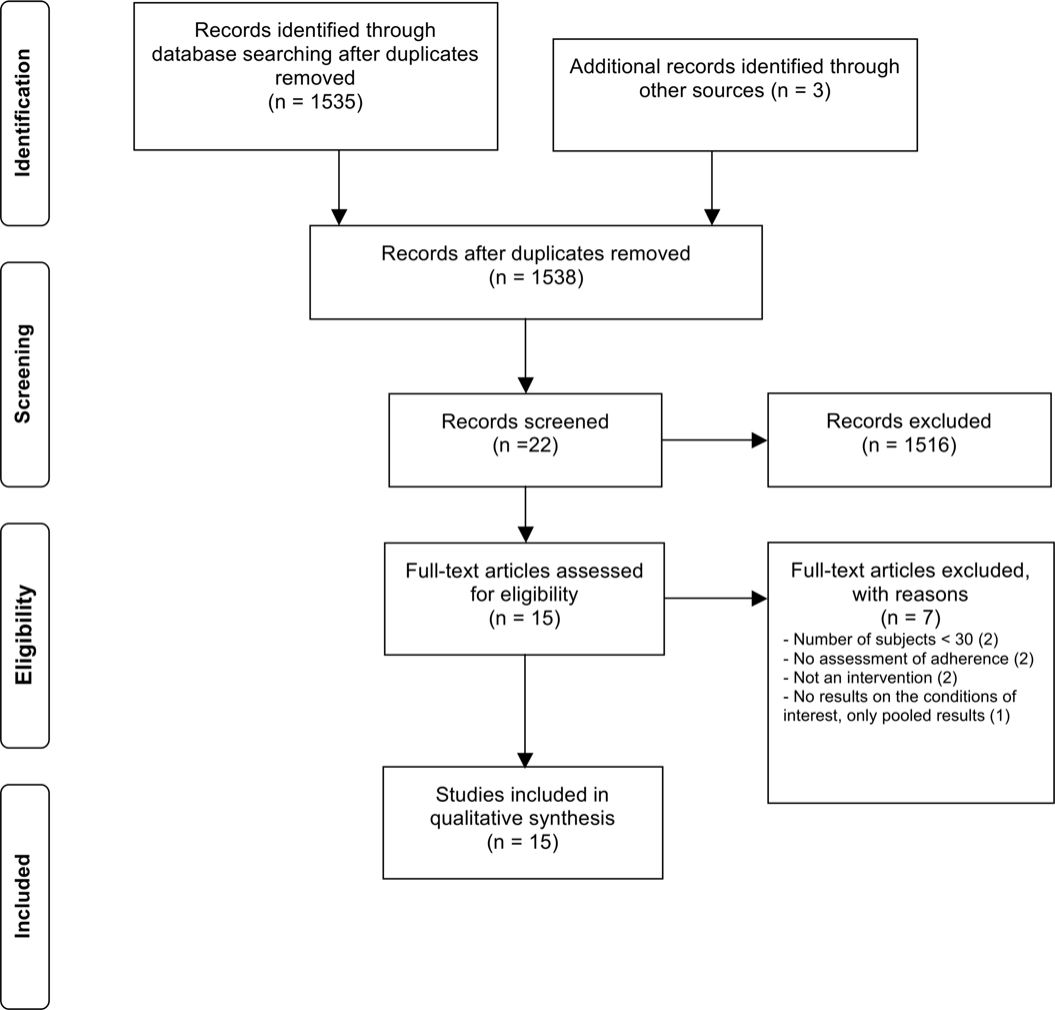
Study selection flow chart.

Of the 1538 citations identified, 22 published articles met the inclusion criteria at the title and abstract review and 15 articles were finally included after full text review (Fig 1). No publication was found in the Cochrane Library or in the reference list of pertinent reviews. All but one studies were randomized controlled trials (RCTs) and one was an observational study. A total of 1958 patients was enrolled in all included studies, 899 with IBD, 579 with RA, 435 with multiple sclerosis, 40 with psoriasis and 0 with SpA. The intervention duration varied from 3 to 18 months.

Forty percent of the studies (6/15) was conducted in patients with IBD [8, 11, 13, 14, 21, 22], half (7/15) in RA patients [10, 12, 15, 23–26], one in psoriasis patients [27] and one in MS patients [28].

Half the interventions (7/15) were classified as multicomponent interventions [8, 11–13, 21, 27, 28], two as behavioral interventions [15, 24], four as educational interventions [10, 22, 23, 26] and two as cognitive behavioral intervention [14, 25].

Considering the data quality, 9 out of 15 studies had no serious limitations on the basis of the GRADE criteria [11–13, 22, 24–28] whereas 6 had serious limitations, mainly because of lack of power [8, 10, 14, 15, 21, 23]. No study was classified as having « very serious limitations » (Tables 3 and 4).

**Table 3:** Summary of evidence in studies with no serious limitations.

Condition	Author	Study design	Type of intervention	Adherence assessment / Magnitude of effect (RR)		*Post-hoc* study power	Risk of bias *
Ulcerative colitis	Elkjaer, 2010	RCT, 12 months	**Multicomponent** (educational + behavioral) *vs*. standard care	- Patient report: Adherence to 4 weeks of treatment was increased by 31% in DK and 44% in Ireland (RR = 1.9 DK; 2.5 Ireland)	✲	DK: 100%	No serious limitations (not validated questionnaire for adherence)
		333 patients		- No effect from prescription database	ø	Ireland: 99%	
	Moshkovska, 2011	RCT, 12 months	**Multicomponent** (educational + cognitive behavioral) *vs*. standard care	- Adherence measured with urinary treatment concentration was greater in the intervention group (RR = 2.4)	✲	97%	No serious limitations
		71 patients					
IBD	Waters, 2005	RCT, 3 months	**Educational** *vs*. standard care	- No significant effect on adherence measured by the mean number of missed medications per month: (difference between groups = 2.52)	ø	93%	No serious limitations
		89 patients					
Psoriasis	Balato, 2013	RCT, 3 months (pilot study)	**Multicomponent** (educational + behavioral) *vs*. standard care	- Adherence to treatment increased in 2.6 days per week In the intervention group whereas no significant variation in the control group in term of days per week in the last week.	✲	No figure to calculate	No serious limitations
		40 patients					
Rheumatoid arthritis	Hill, 2001	RCT, 6 months	**Educational** *vs*. standard care	- Adherence was measured by pharmacological marker		83%	No serious limitations
		100 patients		- At 6 months, 85% of the IG compared with 55% of the CG were taking their medication as prescribed (RR = 1.5)	✲		
	Evers, 2002	RCT, 12 months	**Cognitive behavioral** vs. standard care	- At 12 months, compliance significantly increased in the intervention group (+0.26 on a 3- point scale) while it tended to decrease in the control group	✲	40%	No serious limitations
		59 patients					
	El Miedany, 2012a	Pilot RCT, 12 months	**Behavioral** (visualization of disease progression) *vs*. standard care	- 93% of patients were adherent in the intervention group vs. 70% in the control group (p< 0.01); RR = 1.3	✲	89%	No serious limitations
		111 patients					
	El Miedany, 2012b	RCT, 18 months	**Multicomponent** (educational + behavioral) vs. standard care	- 89% of patients were adherent in the intervention group vs. 64% in the control group (p< 0.01); RR = 1.4	✲	96%	No serious limitations
		147 patients					
Multiple sclerosis	Berger, 2005	RCT, 3 months	**Multicomponent** (educational + behavioral) vs. standard care	-1.2% of patients stopped their medication at 3 months vs. 8.7% in the control group (p< 0.001), RR for adherence = 1.1	✲	95%	No serious limitations
		435 patients					

* According to the GRADE system [19]

✲: significant improvement in the intervention group (IG) vs comparator group (CG)

✱: significant decrease

ø: no significant difference

BMQ: Beliefs about Medication Questionnaire; BSA: Body Surface Area; CCKNOW: Crohn’s and Colitis Knowledge Questionnaire; CQR: Compliance Questionnaire on Rheumatology; DK: Denmark; HAQ-DI: Health Assessment Questionnaire Disability Index; IBD: Inflammatory Bowel Disease; s-IBDQ: Short IBD questionnaire KQ: Knowledge Questionnaire; N/A: Non applicable; PASI: Psoriasis Area Severity Index; PGA: Physician Global Assessment; QoL: Quality of Life; RCT: Randomized clinical trial; RFIPC: Rating Form for IBD Patient Concerns; RR: Relative Risk; SAPASI: Self-administered Psoriasis Area Severity Index; SCCAI: Simple Clinical Colitis Activity Index; SIBDQ: Short Inflammatory Bowel Disease Questionnaire; SIMS: Satisfaction with Information about Medicines Scale.

**Table 4:** Summary of evidence in studies with serious limitations.

Condition	Author	Study design	Type of intervention	Adherence assessment / Magnitude of effect (RR)		*Post-hoc* study power	Risk of bias *
Ulcerative colitis	Cook, 2010	Feasibility trial, 6 months	**Multicomponent** (educational + cognitive behavioral) *vs*. literature data	- Patient report of adherence (defined as months of treatment completed)	✲	80%	Serious limitations (no randomization, no control group, high attrition rate of 51%)
		278 patients		- Participants had higher adherence up to 6 months than the expected rate (RR: 1.5)			
	Cross, 2012	RCT, 12 months	**Multicomponent** (home telemanagement system educational + behavioral) *vs*. standard care	- Adherence measure: MMAS-4			Serious limitations (lack of power due to insufficient recruitment)
		47 patients		- At 12 months, 44% of patients were adherent in the intervention group vs. 68% in the control group (p = 0.10)	ø	36%	
	Moss, 2010	RCT, 6 months	**Cognitive behavioral** *vs*. standard care	- By 6 months, percentage of adherent patients, based on refill data from pharmacies, increased to 67% in the control group vs 50% in the intervention group (p = 0.3)	ø	37%	Serious limitations (lack of power due to effect size lower than expected)
		81 patients					
Rheumatoid arthritis	Van den Bemt, 2011	Mirror image (before-after) study, 6 months	**Behavioral** (report on patient adherence hand to physician) *vs*. standard care	- Adherence measure: CQR			Serious limitations (lack of power)
		50 patients		- No change in adherence after intervention compared to prior intervention **(**p = 0.68)	ø	≤ 10%	
	Homer, 2009	Pilot RCT, 12 months	**Educational** individual *vs*. group counseling	- Pill count: 90% patients counseled in group were adherent vs. 69% patients counseled individually (p = 0.06)	ø	56%	Serious limitations (lack of power)
		62 patients		- On self-reported diaries proportions were similar: group counseling: 97% vs. individual: 94% (p = 1.0)	ø		
	Brus, 1998	RCT, 12 months	**Educational** Experimental group (6 education meeting) vs control group (Brochure on RA)	- After one year, 60% of the patients in the experimental group and 76% in the control group were still using sulphasalazine (p<0.05)	ø	17%	Serious limitations (lack of power)
		55 patients					

* According to the GRADE system [19]

✲: significant improvement in the intervention group (IG) vs comparator group (CG)

ø: no significant difference

BMQ: Beliefs about Medication Questionnaire; CQR: Compliance Questionnaire on Rheumatology; HAQ-DI: Health Assessment Questionnaire Disability Index; MMAS-4: Morisky Medication Adherence Score. QoL: Quality of Life; RCT: Randomized clinical trial; RR: Relative Risk; SCAI: Simple Colitis Activity index; SIBDQ: Short Inflammatory Bowel Disease Questionnaire; SIMS: Satisfaction with Information about Medicines Scale.

### Measure of medication adherence

Adherence was measured on the basis of the declaratives of patients in 8 studies [8, 12, 13, 22, 24, 25, 27, 28]. The others studies (7/15) used a method for which the reliability was tested [10, 11, 14, 15, 21, 23, 26]. This was either a specific validated questionnaire to measure adherence: Morisky Medication Adherence Score or Compliance Questionnaire on Rheumatology [15, 21] or a measure based on data independent from the patient: concentration of medication or metabolites [11, 26], refill data from pharmacies or pill count [10, 14, 23].

### Effect of interventions on medication adherence

Among the studies with no serious limitations, 8 out of 9 studies showed an improvement of adherence [11–13, 24–28], versus 1 out of 6 among the studies with serious limitations [8]. The effective interventions with no serious limitations were conducted in all conditions of interest and were mainly multicomponent interventions with patient education [11–13, 27, 28]. The magnitude of effect as estimated by the RR calculation was moderate ranging from 1.1 to 2.5 in studies with no serious limitations (Table 3).

Among the 6 studies in IBD patients, 3 interventions showed a significant effect on adherence and they were all multicomponent interventions [8, 11, 13]. The 3 other studies in IBD patients had no effect on adherence and represented 3 categories of interventions: educational [22], cognitive behavioral [14], multicomponent [21] (Table 5). Nevertheless, among those 3 studies, two had a very low post-hoc power (Moss et al. and Cross et al. with 37% and 36% respectively), which did not allow to conclude about the effectiveness of these interventions.

**Table 5:** Effectiveness of intervention according to the type of intervention and study limitations.

	Educational	Behavioral	Cognitivo-behavioral	Multicomponent
	n = 4	n = 2	n = 2	n = 7
**Studies with no serious limitations**				
IBD n = 3	ø	-	-	**++**
Rheumatoid arthritis n = 4	**+**	**+**	**+**	**+**
Psoriasis n = 1	-	-	-	**+**
Multiple sclerosis n = 1	-	-	-	**+**
Effectiveness*	1/2	1/1	1/1	5/5
**Studies with serious limitations**				
IBD n = 3	-	-	ø	**+** ø
Rheumatoid arthritis n = 3	ø ø	ø	-	-
Effectiveness*	0/2	0/1	0/1	1/2
**TOTAL EFFECTIVENESS**	**1/4**	**1/2**	**1/2**	**6/7**

ø: negative study

+: positive study (i.e. effective intervention)

*Effectiveness: number of effective studies out of the total number of studies.

In RA, 4 out of 7 interventions showed a significant effect on adherence. These 4 effective interventions corresponded to the 4 different categories of interventions (multicomponent [12], behavioral [24], cognitive behavioral [25] and educational [26]). The 3 others interventions showed negative results. They were two educational interventions [10, 23] and one behavioral intervention [15]. However, these three studies had a dramatic lack of post-hoc power (56%, 17% and 10% respectively).

In psoriasis, the only study included was a multicomponent intervention showed a significant effect on adherence [27]. It was the same thing in multiple sclerosis, with only one multicomponent intervention that showed benefit results on adherence [28].

## Discussion

This systematic review was designed to identify the interventions aimed to improve medication adherence in patients with IMID and to evaluate their effectiveness. Fifteen publications met our eligibility criteria. Whereas the timeframe for study selection was 1990–2013, only five studies were identified before 2010 [22, 23, 25, 26, 28]. This small number illustrates the limited attention to adherence, which is a recent concept in IMID.

Our results support medication adherence interventions as tools to enhance adherence. Nine out of 15 interventions showed an improvement of adherence: three multicomponent interventions in inflammatory bowel disease; one intervention of each category in rheumatoid arthritis; one multicomponent in psoriasis and one multicomponent in multiple sclerosis. We found the strongest evidence for multicomponent interventions (i.e. multiple strategies to enhance adherence) in all conditions of interest. These results are consistent with previous reviews in other chronic conditions such as hypertension, hyperlipidemia, asthma, diabetes or heart failure [29–31]. A meta-analysis of intervention studies on medication adherence published between 1977 and 1994, showed that multidimensional approaches were more effective than unidimensional interventions [30].

Heterogeneity of the published studies did not permit to perform a meta-analysis. In addition, the limited number of studies for psoriasis and multiple sclerosis, for which only one study was identified, needs to be underlined. There is a need to strengthen the evidence regarding the ability of interventions to improve adherence in IMID with well controlled studies.

Despite these limitations, the group of intervention studies identified in this review constitutes a first skeleton from which clinicians and researchers can develop further studies by taking into account the strengths and weaknesses we observed. One major point of improvement should be the quality of study design, especially *a priori* sample size determination to ensure sufficient power to detect a clinically relevant effect. It is noteworthy that 6 out of 15 studies had a dramatic lack of power (<60%). Increasing the power of studies would avoid false negative results, which probably was the case for some of them.

Another point of progress relates to the measure of adherence. Indeed, we observed that a large number of studies (8 out of 15) did not use standardized and validated instruments and are only based on the declaratives of patients. This prevents from comparison between studies and hampers the conduct of meta-analyses. Nonetheless, validated scales exist such as the Morisky Medication Adherence Scale (MMAS, also known as Medication Adherence Questionnaire), which is the first published and the most commonly used adherence scale. [32, 33]. Though there is no gold standard scale for measuring adherence to medication, MMAS is very often recommended because it has a good reliability, is the quick to administer and score and has been validated in the broadest range of diseases [34, 35]. Refill data from pharmacy administrative databases can also be useful for measuring medication adherence, but the analysis of such data need trained researchers to correctly defined and interpreted the adherence indicators [36].

Future research should also attempt to clearly describe all the components of the intervention and to try to identify which specific component is necessary to enhance adherence. Some components may have additive effects, other may have synergistic effects.

The few number of studies identified in this systematic review did not allow to propose recommendations to improve adherence for clinical practice. Nevertheless, our results support the guidelines developed by the National Institute for Health and Clinical Excellence (NICE) regarding medicines adherence and the involvement of patients in decisions about prescribed medicines and supporting adherence [37]. These guidelines indicate that patients need support to improve their medicine taking. This support may take the form of: (i) further information and discussion about the patient beliefs, their concerns and their practical problems about the medicines, and (ii) encouragements to record their medicine taking, to use alternative packaging or a multi-compartment medication device. Because the evidence supporting interventions to increase adherence was inconclusive, the NICE guidelines recommend to consider any intervention to improve adherence on a case by case basis and to tailor the intervention to the specific need of the patient. The recommendations also include advice to healthcare professionals to strenghten communication between the many professionals who may be involved in the individual patient care.

In conclusion, few studies are currently available that evaluate interventions aiming to improve medication adherence in IMID. Although some of them are well-designed, the overall quality of studies could be improved. This regards particularly *a priori* sample size estimation and selection of validated instruments to measure adherence. Nonetheless, the results from this systematic review show that multicomponent interventions appear to be the most efficient in improving adherence to medication.

## Supporting Information

S1 PRISMA Checklist (DOC)
